# Down-Regulation of miR-129-5p and the let-7 Family in Neuroendocrine Tumors and Metastases Leads to Up-Regulation of Their Targets Egr1, G3bp1, Hmga2 and Bach1

**DOI:** 10.3390/genes6010001

**Published:** 2014-12-24

**Authors:** Kristina B. V. Døssing, Tina Binderup, Bogumil Kaczkowski, Anders Jacobsen, Maria Rossing, Ole Winther, Birgitte Federspiel, Ulrich Knigge, Andreas Kjær, Lennart Friis-Hansen

**Affiliations:** 1 Center for Genomic Medicine, Rigshospitalet, Blegdamsvej 9, 2100 Copenhagen, Denmark; E-Mails: kristina@doessing.dk (K.B.V.D.); maria.rossing@rh.regionh.dk (M.R.); 2 Department of Clinical Physiology, Nuclear Medicine and PET, Rigshospitalet, Blegdamsvej 9, 2100 Copenhagen, Denmark; E-Mails: tina.binderup@rh.regionh.dk (T.B.); andreas.kjaer@rh.regionh.dk (A.K.); 3 Cluster for Molecular Imaging, Faculty of Health Sciences, Blegdamsvej 3B, 2100 Copenhagen, Denmark; E-Mail: ulrich.knigge@rh.regionh.dk; 4 The Bioinformatics Center, Department of Biology and Biotech and Research Innovation Centre, Ole Maaløes Vej 5, 2200 Copenhagen, Denmark; E-Mails: b.kaczkowski@gmail.com (B.K.); andersmbj@gmail.com (A.J.); 5 Computational Biology Center, Memorial Sloan-Kettering Cancer Center, 1275 York Ave, New York, NY 10065, USA; 6 DTU Informatics, Technical University of Denmark, Anker Engelunds Vej 1, 2800 Kongens Lyngby, Denmark; E-Mail: ole.winther@gmail.com; 7 Department of Pathology, Rigshospitalet, Blegdamsvej 9, 2100 Copenhagen, Denmark; E-Mail: birgitte.federspiel@rh.regionh.dk; 8 Department of Surgical Gastroenterology C, Rigshospitalet, University of Copenhagen, Blegdamsvej 9, 2100 Copenhagen, Denmark; 9 Department of Clinical Biochemistry Næstved Hospital Ringstedvej 61, 4700 Næstved, Denmark

**Keywords:** neuroendocrine tumors, cancer, miR-129-5p, let-7, EGR1, G3BP1, HMGA2, BACH1, MMP1

## Abstract

Expression of miRNAs in Neuroendocrine Neoplasms (NEN) is poorly characterized. We therefore wanted to examine the miRNA expression in Neuroendocrine Tumors (NETs), and identify their targets and importance in NET carcinogenesis. miRNA expression in six NEN primary tumors, six NEN metastases and four normal intestinal tissues was characterized using miRNA arrays, and validated by *in-situ* hybridization and qPCR. Among the down-regulated miRNAs miR-129-5p and the let-7f/let-7 family, were selected for further characterization. Transfection of miR-129-5p inhibited growth of a pulmonary and an intestinal carcinoid cell line. Analysis of mRNA expression changes identified *EGR1* and *G3BP1* as miR-129-5p targets. They were validated by luciferase assay and western blotting, and found robustly expressed in NETs by immunohistochemistry. Knockdown of EGR1 and G3BP1 mimicked the growth inhibition induced by miR-129-5p. let-7 overexpression inhibited growth of carcinoid cell lines, and let-7 inhibition increased protein content of the transcription factor BACH1 and its targets MMP1 and HMGA2, all known to promote bone metastases. Immunohistochemistry analysis revealed that let-7 targets are highly expressed in NETs and metastases. We found down-regulation of miR-129-5p and the let-7 family, and identified new neuroendocrine specific targets for these miRNAs, which contributes to the growth and metastatic potential of these tumors.

## 1. Introduction

Gastro-Entero-Pancreatic Neuroendocrine Neoplasms (GEP-NEN) are generally slow growing tumors originating from neuroendocrine cells in the gastro-intestinal tract and pancreas [1]. According to the WHO 2010 classification [2] GEP-NEN can be divided into two groups—the well differentiated Neuroendocrine Tumors (NETs) and the poorly differentiated Neuroendocrine Carcinomas (NECs). The NETs are further separated according to proliferative activity (Ki-67 index and mitotic counts) into NET-G1 (equivalent to carcinoid) and NET-G2. The NECs are G3 tumors (NEC-G3) and subtyped into small cell and large cell neoplasms. However, a deeper knowledge about the genetic changes leading to the development of carcinoid tumors is still lacking [1] thereby distinguishing GEP-NENs from the majority of cancer diseases of which there has been a substantial increase in knowledge [3].

One of the hallmarks of cancer cells is their ability to sustain chronic proliferation [4]. Aberrant growth signals independent of growth factor stimuli can be relayed by the RAS-MAPK (Mitogen Activated Protein Kinase) pathway [5]. RAS belongs to a family of small GTPases and acts as a molecular switch cycling between the active GTP-bound—and the inactive GDP-bound form [6]. RAS signaling is attenuated by GTPase activating Proteins (GAPs) [7]. Ras GTPase-activating protein-binding protein 1–3 (G3BP1-3) binds the RasGAP and is important for transducing RAS signals in a MAPK-independent manner [8]. Besides interaction with RASGAP, G3BP1 also has endonuclease activity and seems to regulate stability of mRNAs adding yet another layer of growth control [9,10]. Activation of MAPK increases the expression of the Early Growth Response 1 (EGR1) transcription factor which in turn is linked to key cancer processes such as growth and cell survival [11]. In carcinoids MAPK activation leads to increased tumor cell migration [12]. In breast cancers, inhibition of MAPK suppresses invasion and metastasis in part by increasing let-7 expression, which leads to suppression of the let-7 targets High-mobility group AT-hook 2 (HMGA2) and BTB-and CNC homology 1 leucine zipper transcription factor (BACH1) [13,14].

Growth signals are also controlled by microRNAs (miRNAs) which are short (18–25 nucleotides) non-coding RNAs [15]. miRNAs regulate protein synthesis by either degrading specific mRNA or by translation repression [16]. Because a single miRNA can target multiple mRNAs, one miRNA can regulate several cellular processes [17,18]. Malignant transformation of cells is associated with altered expression of miRNAs [17,18]. We identified two down-regulated miRNAs in NETs and metastases, characterized their actions* in vitro* and identified some of their targets in order to understand how dysregulation of these miRNAs contributes to NET carcinogenesis.

## 2. Experimental

### 2.1. Clinical Samples

Tissues from 9 patients in total with 6 samples from small intestinal NET (G1+G2), 6 samples from metastasis and 4 samples from normal tissue samples (normal tissue was resected between 5–10 cm away from the tumor site) were obtained from patients undergoing surgery for carcinoid tumors at the Department of Surgical Gastroenterology, Rigshospitalet (see Supplementary Table 1 patients 1–9). The inclusion took place from 2008 to 2009 and the study was approved by the regional scientific ethical committee (*journal number* 01 313726) and signed, informed consent was obtained from all participants. Immediately after tumor resection, biopsies were placed in RNA*later^®^* (Applied Biosystems, Carlsbad, CA, USA) for overnight incubation. Samples were subsequently stored at −80 °C until RNA extraction.

One challenge of identifying miRNA differentially regulated between normal gastro-intestinal endocrine cells and gastro-intestinal neuroendocrine tumor/metastasis is obtaining a proper control. Neuroendocrine cells are normally intercalated between the absorptive cells lining the intestines, however, isolating these cells is difficult, and we therefore used normal tissue taken from the same patient from an area close to the tumor site knowing that this may not completely reflect the normal non-malignant cellular processes in the endocrine cells.

### 2.2. Cell Culture

The human pulmonary carcinoid cell line NCI-H727 (ATCC, Manassas, VI, USA) was grown in RPMI-1640 Glutamax (Invitrogen, Carlsbad, CA, USA) supplemented with 10% FBS (Invitrogen), penicillin 100 U/mL and streptomycin 100 µg/mL (Invitrogen), 1 mM Sodium Pyruvate (Invitrogen) and kept at 37 °C with 5% CO_2_. CNDT2 is a human small intestinal carcinoid cell line kindly provided by Lee M. Ellis M.D. Anderson Center Texas USA [19] and kept in DMEM/F12 with 15 mM HEPES (Life Technologies, Carlsbad, CA, USA) supplemented with 10% FBS (Th. Geyer GmbH, Stuttgart, Germany), penicillin 100 U/mL and streptomycin 100 µg/mL (Life Technologies), 5 mL Sodium pyruvate 100 mM (Sigma, St. Louis, MO, USA), 5 mL MEM NEAA 100× (Life Technologies), 5 mL l-Glutamine 200 mM 100× (Life Technologies) and 10 ng/mL NGF (Life Technologies) and kept at 37 °C with 5% CO_2_. The human kidney carcinoma cell line HEK293 (ATCC) was grown in DMEM (Gibco) with 10% FBS (Invitrogen), 100 U/mL penicillin and 100 μg/mL streptomycin (Invitrogen) and incubated at 37 °C with 5% CO_2_. 

### 2.3. RNA Extraction

Total RNA was extracted using Trizol reagent (Invitrogen,) according to the manufacturer’s specifications. The RNA concentration was measured on the NanoDrop (Thermo Fisher Scientific, Waltham, MA, USA) and the RNA integrity was determined using the Agilent 2100 Bioanalyzer (Agilent Technologies, Santa Clara, CA, USA). 

### 2.4. miRNA Microarray Analyses

1200 ng of total RNA from tumors, metastasis or normal tissues were used for labeling per array. For a common reference pool 1200 ng of total RNA from all the tissues together were mixed and hybridized to Invitrogen NCode Multi-Species miRNA Microarray V3 in a Maui hybridization station (Biomicro Systems Inc., Salt Lake City, UT, USA) and run as a two color experiment labeled using Invitrogen’s Ncode Rapid miRNA Labeling System according to the manufacturer’s specifications using the [Cy 3] color reagent for the tissue samples and the [Cy 5] color reagent for the common reference pool. For each run a mix of tumor, metastases and normal tissues were labeled to avoid batch variation. Hybridized slides were scanned in an Agilent DNA microarray scanner (Agilent Technologies) and images of spot intensities were converted to numerical values by GenePix Pro 6.0. The Bioconductor package *limma* was used to normalize the data and to perform the analysis of differential expression. The *p*-values were adjusted for multiple testing using the Benjamini-Hochberg method. Four biological replicates were used for each comparison. Data will be deposited at ArrayExpress upon acceptance. Based on the data obtained from the miRNA arrays, miRNAs for further studies were selected on fold change, *p*-value (*p*-value < 0.05) and knowledge about the miRNAs where available.

### 2.5. qPCR

miRNA expression was assessed using Taqman miRNA assays (Applied Biosystems) for hsa-miR-129-5p, hsa-let-7a and hsa-let-7c. For normalization of the miRNA expression data the geometric mean of hsa-miR-191 and RNU-44 were used [20]. Primer sequences for target validation are listed in Supplementary Table 2.

Reverse transcription reactions are performed using the TaqMan MicroRNA Reverse Transcription Kit (Applied Biosystems) and run on the ABI PRISM 7900 HT Sequence Detection System (Applied Biosystems). 

### 2.6. In Situ Hybridization 

Formalin-fixed and paraffin-embedded (FFPE) tissue samples of 5 carcinoid tumors were obtained from the Department of Pathology (Rigshospitalet, Copenhagen, Denmark). A double-DIG-labeled miR-129-5p LNA (Exiqon, Vedbæk, Denmark) probe sequence GCAAGCCCAGACCGCAAAAAG RNA-T*m* 83 °C was used for detection as described [21]. Sections were counterstained with Nuclear Fast Red.

### 2.7. Immunohistochemistry

Immunohistochemical staining of FFPE slides of 5 NETs (the same as the ones used for miR-129-5p* in situ* hybridization) was performed using primary antibodies against EGR1 (Abcam, Cambridge, UK dilution 1:200) and G3BP1 (Abcam; dilution 1:100). Immunohistochemical staining of paired tumor and metastasis tissue from 5 patients (not the same as for EGR1 and G3BP1, see Supplementary Table 1, patients 10–14) was performed using primary antibodies against BACH1.3 (Abcam; dilution 1:50), MMP-1/8 (Santa Cruz Biotechnology Inc., Santa Cruz, CA, USA dilution 1:50) and HMGA2 (Cell Signaling Beverly, MA, USA, dilution 1:100). Sections were exposed to an antigen retrieval procedure in Target Retrieval Solution High pH (Dako, Glostrup, Denmark) except for HMGA2 where the antigen retrieval was done in Citrate buffer (Sigma) at 98 °C for 15 min, before being incubated with primary antibody for 1 h at room temperature or overnight for HMGA2, followed by secondary antibody (EnVision + Dual Link, Dako) for 30 min at room temperature. After the diaminobenzidine reaction for 10 min at room temperature (liquid DAB + substrate–chromogen system, DakoCytomation), all sections were counterstained with hematoxylin & eosin. 

### 2.8. Transfection Studies and Cell Growth Analyses

Four × 10^6^ NCI-H727 or 150 × 10^3^ CNDT2 cells were seeded and used for each transfection. To 965 µL Opti-MEM (Invitrogen) 10 µL Negative control 1, 2 or siGLO (Thermo Fisher Scientific)/miRNA -miR-129-5p, hsa-let-7-f (Applied Biosystems) hsa-let-7 family inhibitor LNA (Exiqon) or siRNA FlexiTube GeneSolution for EGR1 and siRNA FlexiTube GeneSolution for G3BP1 (Qiagen, Hilden, Germany) is added to a final concentration of 50 nM in 5 mL together with 25 µL Turbofect transfection reagent (Fermentas, Leon-Rot Germany) and left to incubate for 15–20 min at RT before being added drop-wise to the cells. For western blot studies dishes were harvested 12, 24, and 48 h after transfection. For growth analyses 2 × 10^6^ cells were seeded and the media and transfection mix volume was halved. Cells were harvested 24 h after transfection and 40 × 10^3^ cells (NCI-H727) or 15 × 10^3^ cells (CNDT2) were seeded in each well into E-plates for use in the xCELLigence analyzer (Roche/ACEA, San Diego, CA, USA) for proliferation study. Briefly, the xCELLigence analyzer, which is an electronic cell sensor array technology, allows label-free and real-time monitoring of cell proliferation. The presence of the cells on top of the electrodes will affect the local ionic environment at the electrode/solution interface, leading to an increase in the electrode impedance. The more cells are attached on the electrodes, the larger the increases in electrode impedance [22,23]. The difference in cell number seeding for growth assays are due to differences in size and proliferative rate between the two cell lines.

### 2.9. mRNA Microarray Analysis

NCI-H727 and CNDT2 cells were transfected with 50 nM of miR-129-5p duplexes with siGLO siRNA used as a negative control. Total RNA was extracted 24 h post-transfection. Affymetrix microarray analysis HG-U133 Plus 2.0 human (Affymetrix, Santa Clara, CA, USA) was performed at the Microarray Center (Rigshospitalet, Copenhagen, Denmark). Experiments were run in triplicates. Data will be deposited at ArrayExpress. The microarray expression data was processed using the “affy” package in BioConductor [24]. Probe set intensities were summarized and quantile normalized using the BioConductor RMA package. Differential expression was determined per probe set using a *t*-test. The probe sets were mapped to Ensembl transcripts (version 49) using mappings provided at BioMart. Probe sets that mapped to two different Ensembl genes were discarded. 

### 2.10. Evaluating Global Down-Regulation of microRNA Target Genes

The 3’UTRs, 5’UTRs and coding sequences of the transcripts were scanned for matching 6mer, 7mer and 8mer miRNA seed sites (complementary to position 2–7, 2–8, and 2–9 of the miRNA). Global analysis of miRNA target down-regulation was evaluated using the longest 3’UTR sequence per gene to avoid bias introduced by genes with many transcript isoforms. We discarded transcripts with 3’UTR sequences shorter than 50 nt. To globally evaluate if miRNA target genes were down-regulated after miRNA transfection, we tested the null hypothesis that the expression change distribution of miRNA targets (having a 7mer target site) were equal to the distribution of all expressed genes without predicted target sites using the non-parametric Wilcoxon rank-sum test. A similar approach was used to evaluate down-regulation of genes with miRNA target sites in coding regions and 5’UTRs of mRNAs.

### 2.11. Statistical Assessment of 3'UTR Words Correlated with mRNA Down-Regulation

We used a previously published non-parametric rank-based statistic to assess the correlation of word occurrences in 3’UTRs and the change in gene expression after miRNA transfection [25,26]. Genes were sorted by expression change induced by transfection of miR-129-5p and the correlation with down-regulation was tested for all words of length 5–7 (*N* = 21 504).

### 2.12. Gene Set Enrichment Analysis

We examined if the genes differentially expressed upon miR-129-5p transfection were enriched in specific GO, KEGG or PFAM terms. The lists of genes with Benjamini-Hochberg adjusted *p*-values below 0.05 were checked for enrichment using *GOstats* package from Bioconductor.

### 2.13. 3'UTR Luciferase Assay

The 3'UTR for Egr1 and G3bp1 were amplified from human genomic DNA and cloned into the pmiR-report luciferase reporter plasmid generating pmiR-report-*EGR1*—3'UTR and pmiR-report-*G3BP1*-3'UTR. Plasmids containing mutated seed recognition sequence were subsequently generated. To exclude the actions of any endogenous miRNAs the assay was performed in the HEK293 cell line (ATCC) since the overall content of miRNAs in this particular cell line is very low [27]. One hundred × 10^3^ cells were seeded in 24 well plates and transfected the next day after standard procedure with a transfection mix consisting of 100 ng pmiR construct, 6.25 ng Renilla, 1 µL Turbofect, 1 µL pre-miR to a final concentration of 5 nM and Optimem ad 100 µL. The next day cells were lysed in Passive Lysis Buffer (Promega, Madison, WI, USA), vortexed 30 min and centrifuged 10 min. Thirty µL supernatants were transferred to white 96 well plates and signals from the plasmid constructs were measured and analyzed by using the Dual-Luciferase Reporter Assay System (Promega) and the Glomax 96 micro plate luminometer (Promega) according to the manufacturer’s specifications.

### 2.14. Western Blotting

Samples were run on a Clear Page 4%–20% denaturing gel (C.B.S. Scientific, San Diego, CA, USA). By using the iBlotting (Invitrogen) system, proteins were transferred to a membrane and blocked in 5% w/v milk/0.01% w/v Tween-20/PBS incubated with primary antibody, either EGR1 ab55160, G3BP1 ab39533 or BACH1 ab54814 (Abcam), MMP1 sc30069 (Santa Cruz Biotechnology Inc., Dallas, TX, USA), HMGA2 8179 (Cell Signaling) or Anti-Histone H3 05-499 (Millipore, Billerica, MA, USA) overnight and incubated with secondary antibody for 1 h. ECL Select^TM^ Western Blotting Detection Reagent (GE Healthcare Life Sciences, Uppsala, Sweden) was added to the membrane for 2 min. The luminescent signal present on the membrane was captured by a Fuji Film Las1000 Luminescent Image Analyzer Model LAS1000plus IDX2 using the Image Reader LAS1000 Pro v.2.5 and Image Gauge v.4.0 software captured the luminescent signal present on the membrane and was used for signal quantification. All western blots were repeated 3–5 times and with similar results When transfecting cells with a miRNA the target was down-regulated by quantification or up-regulated when transfecting cells with a miRNA-inhibitor. Only a representative blot is shown.

### 2.15. Statistical Analyses

Students’ unpaired *t*-test was used and differences with a *p* ≤ 0.05 were considered significant and indicated by *. Unless otherwise stated results are given as median ± SD.

## 3. Results

### 3.1. miR-129-5p is Down-Regulated in Neuroendocrine Tumors and the let-7 Family is Down-Regulated in Neuroendocrine Tumor Metastases

We used miRNA array to identify miRNAs that are differentially regulated in tumors and metastases. Comparing NET primary tumors and metastases to the adjacent normal tissues we found that 48 out of a total of 1174 miRNAs were differentially expressed. Of these, 18 were down-regulated and 30 up-regulated (*p*-value < 0.05) (Supplementary Table S3). Based on fold-changes and *p*-values of the down-regulated miRNAs, miR-129-5p was selected for further analysis (Table 1). 

**Figure 1:**
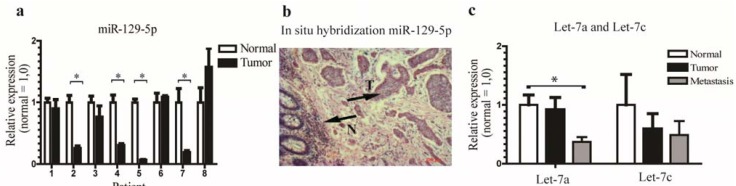
The expression of miR-129-5p and let-7 are down-regulated in neuroendocrine tumors. (**a**,**b**) qPCR analysis and *in situ* hybridization showed little expression of miR-129-5p in NETs compared to the adjacent normal tissue (scale bar 100 µm) consistent with the qPCR results. Arrows with N and T denote Normal and Tumor tissue respectively. The average expression of the miRNAs is normalized to the geometric mean of RNU44 and miR-191; (**c**) qPCR analysis of let-7a and let-7c showed a clear down-regulation in tumors compared to matched control tissue and metastases compared to tumors respectively, although the down-regulation was not significant for let-7c.* = *p* < 0.05.

To associate miRNA changes with tumors and metastasis, we compared metastases and normal tissues and metastases and tumor tissues. In metastasis* vs.* normal tissue we found 145 miRNAs differentially expressed—77 down-regulated and 68 up-regulated (*p*-value < 0.05) (Supplementary Table 3). In tumor* vs.* metastasis 163 differentially expressed miRNAs—80 down-regulated and 83 up-regulated (*p*-value < 0.05) were identified. Noticeably, 8 members of the let-7 family (let-7 –a, -b, -c, -d, -e, -f, and -i) were down-regulated in both comparisons, underlining the let-7 family’s role in metastasis (Table 1). To validate the reduced expression in carcinoids we performed qPCR on independent samples from laser capture micro dissected tissue from blocks from a different patient group also diagnosed with NETs and *in-situ* hybridization analysis for miR-129-5p (Figure 1a,b) and for the let-7 family (Figure 1c) on the same patient group as the qPCR. 

**Table 1:** Differentially regulated miR-129-5p and let-7.

T + M* vs.* N	Log FC	FC	*p*-value
hsa-miR-129-5p	−1.3	0.4	0.01
hsa-let-7a	−1.7	0.3	0.002
hsa-let-7c	−1.7	0.3	0.001
*hsa-let-7b*	*−1.5*	*0.3*	*0.0003*
*hsa-let-7d*	*−1.5*	*0.3*	*0,003*
*hsa-let-7e*	*−1.4*	*0.4*	*0.002*
*hsa-let-7f*	*−1.4*	*0.4*	*0.003*
*hsa-let-7g*	*−1.1*	*0.5*	*0.01*
*hsa-let-7i*	*−1.3*	*0.4*	*0.001*
M* vs.* N	Log FC	FC	*p*-value
hsa-let-7a	−1.7	0.3	0.002
hsa-let-7c	−1.7	0.3	0.001
*hsa-let-7b*	*−1.5*	*0.3*	*0.0003*
*hsa-let-7d*	*−1.5*	*0.3*	*0.003*
*hsa-let-7e*	*−1.4*	*0.4*	*0.002*
*hsa-let-7f*	*−1.4*	*0.4*	*0.003*
*hsa-let-7g*	*−1.1*	*0.5*	*0.01*
*hsa-let-7i*	*−1.3*	*0.4*	*0.001*
M* vs.* T	Log FC	FC	*p*-value
hsa-let-7a	−0.03	0.8	0.3
hsa-let-7c	−0.02	1.0	0.9
*hsa-let-7b*	*−0.3*	*0.8*	*0.3*
*hsa-let-7d*	*−0.2*	*0.9*	*0.6*
*hsa-let-7e*	*−0.1*	*0.9*	*0.6*
*hsa-let-7f*	−*0.1*	*0.9*	*0.8*
*hsa-let-7g*	−*0.1*	*0.9*	*0.8*
*hsa-let-7i*	−*0.02*	*1.0*	*0.9*

miRNAs are chosen on the basis of fold change and *p*-value. Only miR-129-5p and let-7a and c have been verified by qPCR analysis, the rest of the let-7 family members are included to show that the whole family is lost. T + M* vs.* N and T* vs.* M denote Tumor and Metastasis* vs.* Normal tissue and Tumor* vs.* Metastasis respectively.

### 3.2. miR-129-5p Inhibits Growth of the Carcinoid Cell Lines NCI-H727 and CNDT2

**Figure 2:**
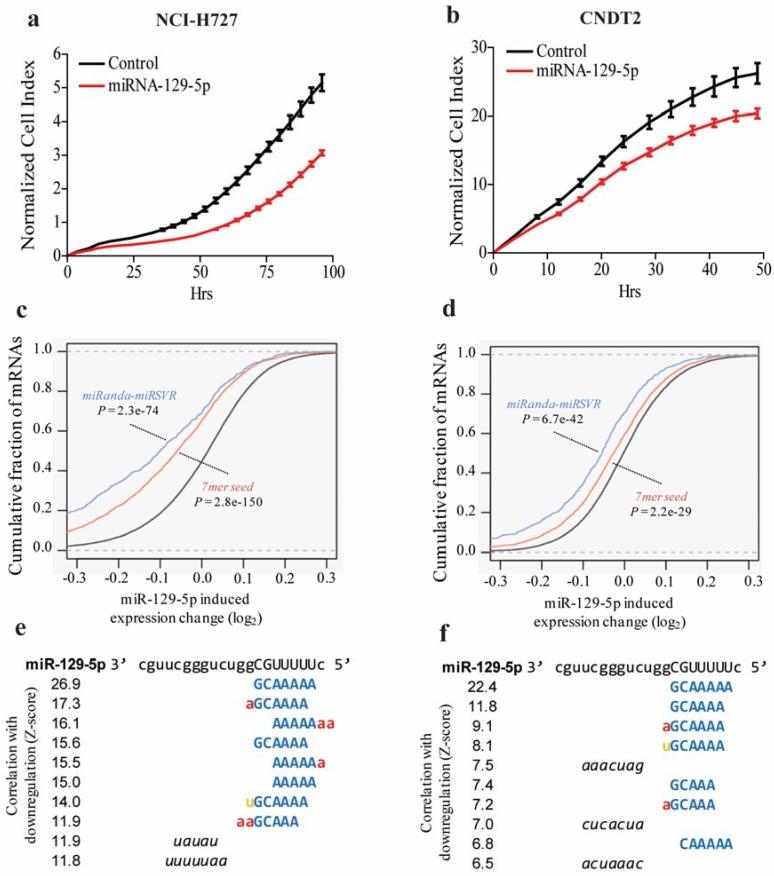
miR-129-5p inhibits growth of carcinoid cell lines. (**a**,**b**) NCI-H727 and CNDT2 cells were transfected with miR-129-5p or a control and the growth was examined using the xCELLigence system. A marked growth inhibition is seen when cells are transfected with miR-129-5p. For NCI-H727 cells the slopes are 5.6 × 10^−2^ h^−1^ (Control) compared to 3.7 × 10^−2^ h^−1^ (miR-129-5p). For the CNDT2 cells the slopes are 6.0 × 10^−1^ h^−1^ (Control) compared to 4.6 × 10^−1^ h^−1^; (**c**–**f**) The global changes in gene expression in NCI-H727 and CNDT2 cells respectively following transfection of miR-129-5p were determined by microarray. Subsequent word analysis of the down regulated transcripts showed an enrichment of transcripts containing the 7-mer targets of words.

NETs usually occur in the gastro-intestinal tract and lung [28], we therefore used both a pulmonary carcinoid cell line, NCI-H727, and a small intestinal carcinoid cell line, CNDT2, as model systems for NETs and for* in vitro* investigation of the effect of the identified miRNAs. Given that growth is one of the hallmarks of cancer [4] we examined how over-expression of miR-129-5p affected the growth of cells* in vitro* and found that it inhibited the growth of both of the pulmonary carcinoid cell line NCI-H727 and the small intestinal CDNT2 carcinoid cell line (Figure 2a,b) indicating that both cell lines are good model systems for NETs and that at least some of the biological processes determining growth in these cell lines are identical. 

To further describe the biological actions of miR-129-5p, we characterized its mRNA targets. We found that mRNAs with predicted miRNA target sites (7mer seed site) in the 3’UTR were significantly down-regulated compared to mRNAs without predicted target sites after transfection of miR-129-5p in both cell lines (p = 5.5e–23, two-tailed Wilcoxon rank-sum test) (Figure 2c,d). Unbiased word analysis of all oligonucleotides (words) of length 5–7 demonstrated that the miR-129-5p 7mer seed site was the 3’UTR word most correlated with down-regulation in the cell lines (Figure 2e,f). Gene Ontology (GO) term enrichment analysis showed that miR-129-5p mainly controlled pathways related to nucleotide metabolism and RNA binding in the NCI-H727 cell line (data not shown).

### 3.3. miR-129-5p Targets EGR1 and G3BP1 in Carcinoid Cell Lines NCI-H727 and CNDT2

**Figure 3:**
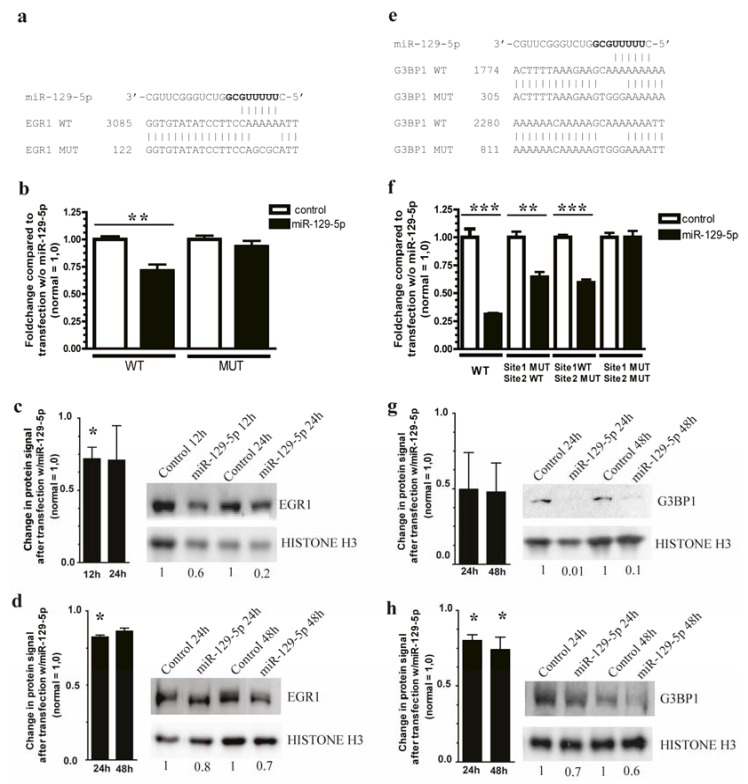
*EGR1* and *G3BP1* are targets of miR-129-5p. (**a**,**e**) Direct interaction between the miR-129-5p and its target sites in *EGR1* and *G3BP1* 3’UTRs was determined using luciferase assays. The miR-129-5p sequence is followed by the wild type EGR1 and G3BP1 3’UTR target sequences. At the bottom the mutated target sites are shown with changes in the 4 central nucleotides that should abolish binding of miR-129-5p to the two targets; (**b**,**f**) Transfection of miR-129-5p significantly reduced the luciferase activity of constructs containing the wild type but not mutated 3’UTRs constructs, ** *p* < 0.01, *** *p* < 0.001 (**c**,**d**,**g**,**h**) Transfection of NCI-H727 (**c**,**g**) and CNDT2 (**d**,**h**) cells respectively with miR-129-5p leads to a reduction of EGR1 and G3BP1. Protein signals are quantified and values are below each lane, only representative blots are shown and densitometry below each lane represents that blot. The bars represent the combined densitometry results for the western blot replicates, and statistic significances, if any, are indicated above.

EGR1 and G3BP1 were among the most down-regulated targets following miR-129-5p over-expression in both the NCI-H727 and CNDT2 cell line and were therefore selected for further analyses (Supplementary Table S4). *EGR1* is a transcription factor [29] and *G3BP1* is involved in both RNA metabolism and Ras activation [9]. There is one target sequence for miR-129-5p in the 3'UTR of EGR1 and two in that of G3BP1. Direct interaction of miR-129-5p with the target gene 3' UTRs was demonstrated by luciferase assay and showed a significant reduction in signals. For G3BP1 miR-129-5p binding to the distal target sequence leads to a greater inhibition of the luciferase signal than binding to the proximal target sequence, and both sites acted synergistically. The finding that the most effective site resides near the end of the 3’UTR of G3BP1 and that both sites were associated with greater mRNA destabilization in our transfection experiments has also been observed for other mRNAs [30] (Figure 3a,b,e,f). 

Western blot analyses confirmed that the expression of the EGR1 and G3BP1 proteins were reduced after re-introduction of miR-129-5p to both carcinoid cell lines (Figure 3c,d,g,h). This confirms both EGR1 and G3BP1 as new bona fide miR-129-5p targets.

### 3.4. EGR1 and G3BP1 are Up-Regulated in Neuroendocrine Tumors and Promote Growth in Carcinoid Cell Lines

**Figure 4:**
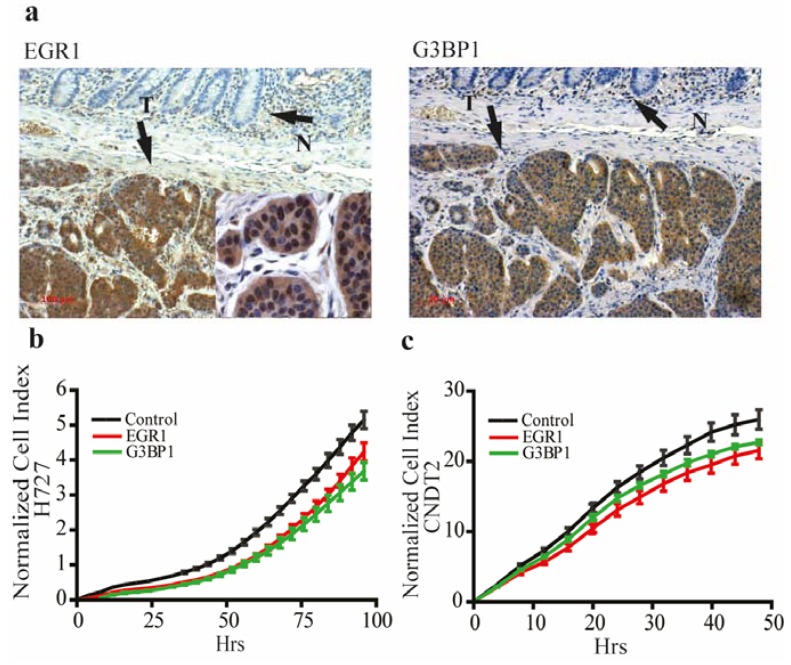
EGR1 and G3BP1 are present in neuroendocrine tumors and important for the growth in a carcinoid cell line. (**a**) The two miR-129-5p targets *EGR1* and *G3BP1* are robustly expressed in NETs compared to the adjacent normal tissue where expression of the two targets are significantly lower as shown by immunohistochemistry. Immunohistochemistry was performed on all the tumors used in the qPCR analysis for miR-129-5p, for simplicity only one is shown here (scale bar 100 µm) EGR1 insert shows nuclear staining for EGR1 in NETs shown in 600× magnification; (**b**,**c**) siRNA knock down of either EGR1 or G3BP1 markedly inhibits the growth of the NCI-H727 cells and CNDT2 cells respectively. The slopes of the growth curves are for NCI-H727 4.64 × 10^−2^ h^−1^ (control) compared to 3.58 × 10^−2^ h^−1^ (EGR1) and 3.30 × 10^−2^ h^−1^ (G3BP1). When looking at the CNDT2 cell line the slopes of the growth curves are 5.68 × 10^−1^ h^−1^ (Control) compared to 4.67 × 10^−1^ h^−1^ (EGR1) and 4.96 × 10^−1^ h^−1^ (G3BP1).

Given the reduced expression of miR-129-5p in NETs we examined the expression of *EGR1* and *G3BP1* in NETs and we found the two genes highly expressed in NETs by immunohistochemistry (Figure 4a). 

To characterize the biology of *EGR1* and *G3BP1* in NETs, we examined their importance for growth of carcinoid cell lines. We found that knock down of EGR1 and G3BP1 inhibited the growth of carcinoid cell lines compared to controls (Figure 4b,c). This suggests that at least some of miR-129-5p’s biological actions in NETs is exerted though control of *EGR1* and *G3BP1*.

### 3.5. The let-7 Family is Involved in the Neuroendocrine Tumor Metastatic Process by Targeting HMGA2 and BACH1

**Figure 5:**
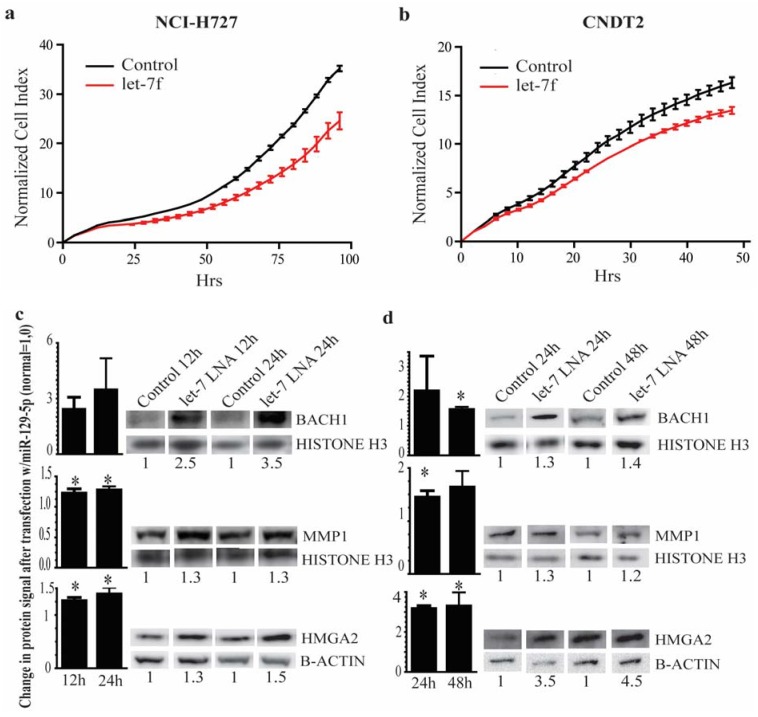

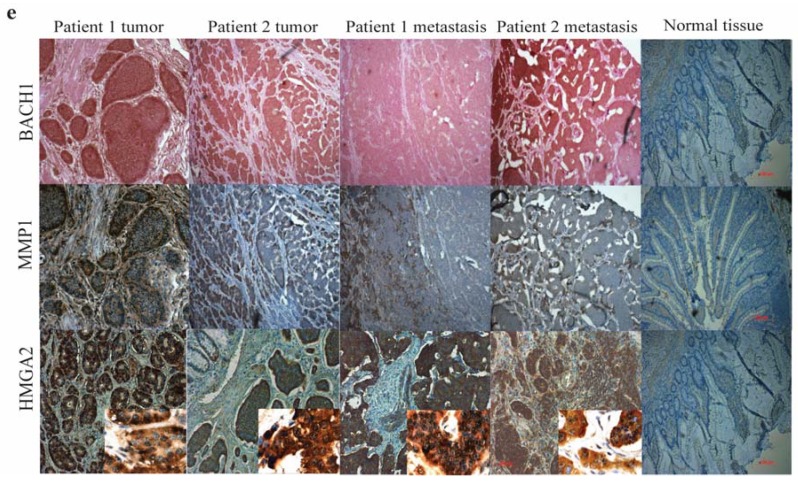
let-7 inhibits growth of a carcinoid cell lines and targets genes involved in the metastatic event. (**a**,**b**) NCI-H727 and CNDT2 cells respectively were transfected with let-7f or a control and the growth was examined using the xCELLigence system. A marked growth inhibition is seen when cells are transfected with let-7f. For NCI-H727 cells the slopes are 5.6 × 10^−2^ h^−1^ (Control) and 3.66 × 10^−2^ h^−1^ (let-7f); for the CNDT2 cells the slopes are 3.3 × 10^−1^ h^−1^ (Control) and 2.7 × 10^−1^ h^−1^ (let-7f); (**c**,**d**) Transfection of NCI-H727 and CNDT2 cells with a let-7 family inhibitor leads to a marked increase in BACH1, MMP1 and HMGA2 protein signals (see the quantified values below each lane). Only representative blots are shown and densitometry below each lane represents that blot. The bars represent the combined densitometry results for the western blot replicated and statistic significances, if any, are indicated above; (**e**) Immunohistochemistry of NETs and paired metastasis show a robust expression of BACH1, MMP1 and HMGA2, for comparison immunohistochemistry for BACH1, MMP1 and HMGA2 was also performed in normal intestine far right panel (scale bar 100 µm). HMGA2 inserts show nuclear staining for HMGA2 in NETs shown at 600× magnification.

Almost half of the patients with NETs have regional or distant metastases at the time of diagnosis [31]. *HMGA2* is a chromatin remodeling factor and *BACH1* is a transcription factor which transcribes the Matrix Metalloproteinase 1 (*MMP1)*. All three acts synergistically to induce bone metastasis and HMGA2 and BACH1 are key targets for let-7 inhibition and highly involved in the metastatic process [14,32]. We therefore examined how re-introducing the expression of a let-7 family member affected the growth of carcinoid cell lines. Transfection with let-7f inhibited the growth of both the carcinoid cell lines (Figure 5a,b). 

Secondly we examined the biological consequence of reduced let-7 expression in NETs and metastasis by western blot analyses and immunohistochemistry on paired FFPE tumor and metastases tissues for HMGA2, BACH1 and MMP1. Western blot analysis showed an increase in cellular content of the HMGA2, BACH1 and MMP1 proteins (we assume that the modest increase seen in MMP1 in comparison to BACH1, might be due to other regulatory processes in the different tissues and cell lines than that of BACH1) when a let-7 family inhibitor was transfected into the carcinoid cell lines confirming the two proteins as genuine targets of let-7 (Figure 5c,d). The immunohistochemistry showed a robust expression of the three proteins in the NET tumor and paired metastatic tissues (Figure 5e), which, to the best of our knowledge, is the first time all three proteins have been demonstrated in neuroendocrine tumors and their metastases.

## 4. Discussion

We have shown that the expression of miRNAs is deregulated in small intestinal NETs (G1+G2) and found indication that the number of de-regulated miRNAs seems to increase as the tumors metastasize. Our observation parallels the report by Lloyd and colleagues who recently also found aberrant miRNA expression in NETs [33]. They examined 95 miRNAs and we found some of the same, but not all, to be down- or up-regulated. The difference between our findings and those of Lloyd and colleagues can be attributed to the difference in the miRNA included in the different assays and technical differences between the platforms used (Invitrogen miRNA microarray, this study) and the Cancer MicroRNA qPCR Array Kit [33,34,35]. It could also be attributed to the relatively small number in sample size in our experiments. Robust bioinformatics is best done on paired samples and a larger group of samples; however we only had access to limited material. Therefore we carefully validated our results both in cell lines and in neuroendocrine tissue from patients. In addition miR-129-5p and the let-7 family are known to have an anti-proliferative and anti-metastatic effect in other cancers which makes them even more likely to be missing in neuroendocrine cancers [13,36]. 

Access to suitable* in vitro* and* in vivo* model systems for carcinoid tumors are important for examining the biological impact of observations made from clinical samples. We found that that in terms of the response to miR-129-5p and let-7f manipulation both model cell lines (NCI-H727 and CDNT2) responded in a similar manner. Hence, we consider them good* in vitro* models of NET biology.

We focused on miR-129-5p, which we found to be down-regulated in NETs. We found that transfection of miR-129-5p inhibited carcinoid cell growth. miR-129-5p has also been found down-regulated in gastric cancer [37], bladder cancer [38] and colorectal cancer [39]. In these tumors miR-129-5p was also shown to inhibit growth consistent with miR-129-5p being a tumor suppressor miRNA [40]. Global analysis of pathways affected by miR-129-5p showed that it primarily targeted RNA binding and nucleotide metabolism. We identified *EGR1* and *G3BP1*, both known to regulate these processes as novel targets for miR-129-5p.

In some cancers *EGR1* inhibits growth [41] where as in others *EGR1* is associated with tumor progression, for instance in prostate and gastric cancer which possess endocrine components [42,43]. We found increased expression of EGR1 in the NETs and that knockdown of *EGR1* inhibited growth of the carcinoid cells. Recently, Edtfeld* et al.* have associated increased *EGR1* expression with NET progression [44]. The transcription of *EGR1* is controlled by the MAPK signaling pathway through phosphorylation and activation of transcription factors of the ETS like transcription factor (ELK-1) family by Extracellular-signal-Regulated Kinases (ERK1/2), p38MAPK and/or c-Jun *N*-terminal kinases (JNK) [45,46]. A particular feature of NENs are that they have elevated levels of Ras-related Protein 1 (RAP1) and Serine/threonine-protein kinase B-raf (B-RAF) [47] which in turn activates MAPK thereby enabling the high expression levels of *EGR1* leading to increased tumor progression.

The Extracellular-signal-regulated kinases (ERK) pathway can be activated by growth factors such as Epidermal Growth Factor (EGF) [48], and when activated by ERK and a subsequent induction of *EGR1* exceeds a critical threshold, cells are permitted to enter the S phase and divide [49]. Thus, there seems to be a “digitization” of the biological response to a graded EGR1 expression signal. Although miRNAs mainly modulate gene expression, a reduction in miR-129-5p could be important for this mechanism as it would result in increased amounts of EGR1. Modulation of these signals may be important for neuroendocrine carcinogenesis as for instance Epidermal Growth Factor Receptor (EGFR) signaling is important for neuroendocrine growth [50,51].

Moreover, we found G3BP1 to be a novel target of miR-129-5p. *G3BP1* is over-expressed in NETs, which in this respect resemble other tumors such as colon, thyroid, breast, lung, and head-neck tumors [52]. Loss of *G3BP1* leads to growth retardation [53] whereas over-expression promotes growth and migration [9,54]. G3BP1 acts as an effector of RAS as it only binds active RAS, thereby contributing to the signaling cascade via MAPK [55]. In many cancers mutations that activates *RAS* are driven by mutations in BRAF [56] but BRAF mutations are rare in NETs [57]. However, the activating protein RAP1 is frequently expressed and the Raf proto-oncogene serine/threonine-protein kinase (RAF1) RAF1/BRAF signaling pathway is activated in NETs [47] making this pathway a putative therapeutic target. We showed that by inhibiting *G3BP1* we can inhibit growth of carcinoid cells demonstrating that indeed this pathway is important in neuroendocrine carcinogenesis. Because miR-129-5p targets both G3BP1 and EGR1 we propose, that miR-129-5p is an important regulator of oncogenic signals in small intestinal NETs. 

We found the expression of many of the let-7 family members down-regulated in NETs metastases. The let-7 family consists of 13 family members [58] that are important for promoting cell-cycle exit and terminal differentiation [59]. In some cancers all or most members have been reported lost or down-regulated e.g., gastric cancer, lung cancer, and head and neck cancers [60]. In other cancers only a few specific members have been shown to be lost e.g., let-7b in melanoma [61]. Metastatic NENs seem to belong to the first group, though the biological differences between down-regulation of many or few let-7 members is poorly understood. We found that transfection of let-7f inhibited growth of carcinoid cells and that many of the let-7 targets involved in the metastatic process was up-regulated. In NENs and their metastases we found increased expression of *HMGA2*, *BACH1* and its target *MMP1*. Some of the changes in miRNA expression associated with the metastatic process in NETs are similar to what has been seen when other tumors metastasize. Reduced expression/loss of let-7 has also been seen when for instance breast cancer [14], gastric cancer [62], colorectal cancer [63], and renal cancer metastasize [64]. *HMGA2* over expression in itself is a hallmark for both benign and malignant tumors and also linked to a highly malignant phenotype with a poor prognostic index. The importance of particularly the let-7 family of miRNAs in suppressing *HMGA2* is evident by the fact that multiple let-7 binding sites are in the 3’UTR of *HMGA2*. The correlation between let-7 down-regulation and *HMGA2* over-expression in NETs previously shown by Rahmann* et al.* [60] suggests that the basis for metastasis is founded very early in the carcinogenesis of NETs and lasts until the tumor actually metastasizes. In addition there is a correlation between low let-7 expression in tumors with high *HMGA1/2* expression and the grading stages of NETs where the high grade and highly invasive tumors also have high expression of *HMGA1/2* and low let-7 expression. The same relationship is also seen with respect to tumor size [60,65].

The anti-tumorigenic effect of let-7 by targeting both *HMGA2* and *BACH1* is especially effective in abrogating bone metastasis in a breast cancer model where the two proteins share some but not all of the same target genes. Knocking down both *BACH1* and *HMGA2* suppressed metastatic invasion, homing and osteolysis [32], where knocking down only one of the two proteins did not suppress enough of the target genes in these pathways to prevent metastasis. In the past bone metastasis originating from NETs was considered rare since they usually metastasize to the liver, lymph node and lungs, however, they are becoming more and more prevalent due to both better and earlier detection and because of the longer survival of NET patients [28]. The mechanism behind bone metastasis from NETs has as of yet not been fully elucidated, but the cooperation between some of the same key players, as in the breast cancer model, seems a definite possibility based on our results. The importance of recognizing the fact that NETs do metastasize to the bones lies in the fact that this can be used as a prognostic factor for overall survival [28].

## 5. Conclusions

In conclusion we have shown that miRNAs are dysregulated in NETs and identified two of the down-regulated miRNAs in NETs, miR-129-5p and the let-7 family to have growth inhibitory effects. We identified two new targets EGR1 and G3BP1 for miR-129-5p and discovered a possible pathway for bone metastasis involving let-7 and its targets HMGA2 and BACH1.

## Supplementary Files

Supplementary File 1
