# Supplementary material for: Down-Regulation of miR-129-5p and the let-7 Family in Neuroendocrine Tumors and Metastases Leads to Up-Regulation of Their Targets Egr1, G3bp1, Hmga2 and Bach1
Source: Genes (Basel). 2014 Dec 24;6(1):1–21. doi: 10.3390/genes6010001 (PMC4377830; doi:10.3390/genes6010001)
Supplement: Supplementary File 1 [file genes-06-00001-s001.zip › genes-67534-supplementary/genes-67534-Suppl Tables 1 & 2.doc]

**Supplementary Table 1.** Tissue used for miRNA microarray analysis and let-7 target immunohistochemistry.

| **Patient** | **Age/Sex** | **Tissue** * | **Location** | **Ki67 index** |
| --- | --- | --- | --- | --- |
| **1** | 72/M | T + M + M | Ileocecal junction, Liver and Lymphnode | <2%T |
| **2** | 59/M | M | Retroperitoneal | 8% |
| **3** | 69/F | T | Small intestine | <2% |
| **4** | 62/F | N + T + M | Small intestine, small intestine and Liver | <1%T <2%M |
| **5** | 61/M | N + T | Colon and Ileocecal junction | <2%T |
| **6** | 75/M | M | Lymphnode | 5% |
| **7** | 69/F | N + T + M | Coecum, coecum and Lymphnode | 85%M |
| **8** | 66/M | T | Colon | <1% |
| **9** | 68/F | N | Small intestine | - |
| **10** | 65/F | T + M | Colon and Liver | 1% T, 2%M |
| **11** | 69/F | T + M | Small intestine and Liver | 3% T |
| **12** | 62/M | T + M | Ileum and Lymphnode | 2% T |
| **13** | 55/F | T + M | Small intestine and Lymphnode | 7%–8% T |
| **14** | 64/F | T + M | Ileocecal and Lympnode | 1% T |

* N, T and M denotes Normal, Tumor and Metastasis tissue respectively, where possible the Ki67 index
is indicated. Patients 1–9 are used for miRNA microarray analysis and 10–14 for immunohistochemistry, patient 10 and 14 are used for Figure 5. See text for details.

**Supplementary Table 2.** qPCR primers.

| **MiRNA** | **Mature Sequence** | **Species** | **Product ID** |
| --- | --- | --- | --- |
| miR-129 | CUUUUUGCGGUCUGGGCUUGC | hsa | 000590 |
| Let-7-a | UGAGGUAGUAGGUUGUAUAGUU | hsa | 000377 |
| Let-7-c | UGAGGUAGUAGGUUGUAUGGUU | hsa | 000379 |
| MiR-191 | CAACGGAAUCCCAAAAGCAGCUG | hsa | 002299 |
| RNU-44 | CCTGGATGATGATAGCAAATGCTG-ACTGAACATGAAGGTCTTAATTAGCTCTAACTGACT | hsa | 001094 |
